# Schools of Massage.—II. St. Thomas's Hospital Physical Exercises and Massage School

**Published:** 1916-03-25

**Authors:** 


### SCHOOLS OF MASSAGE.

### II. St. Thomas's Hospital Physical Exercises and Massage School.

The physical exercises and massage department and training school at this hospital has only existed in its present form for four years. A small department for curative physical treatment was initiated in 1898, but the number of patients dealt with was not great. In 1906 Dr. Timberg, M.R.C.S., L.R.C.P., graduate of the Royal Gymnastic Institute, Stockholm, was invited by the hospital authorities to undertake the work of developing the department on lines that he had seen successfully carried out in his own country, with the result that at the present time this branch of work has become one of the leading departments of the hospital. The work of evolving such an embryonic department to the important and complete organisation which now exists in the short space of four years speaks volumes for the steady and persistent efforts made by the medical superintendent, for, as the number of patients increased, more space for their treatment and improved equipment were gradually added, until in 1911 it was decided to unite physical exercises, massage treatment (which till then had not been under Dr. Timberg's supervision), and the electrical section in one group.

A ward on the ground floor was adapted to provide accommodation for the extended work of this department, and, in order to provide workers to deal with the very large number of patients under treatment, a training school was organised to accommodate twenty-four lady students who, though residing in the hospital and being under the control of the matron, would be quite apart from the nursing staff. The immediate control of the students was placed in the hands of Miss Randell, who has been a ward sister, and is therefore familiar with the ways of the hospital. She also holds the certificate of the Incorporated Society of Trained Masseuses and the Teacher's Diploma for Massage and Remedial Exercises.

The full course of massage, remedial exercises, and electricity occupies a period of eighteen months, and the pupils are prepared for the examination of the Incorporated Society of Trained Masseuses in these subjects. The course includes educational gymnastics, anatomy, physiology, theory of movement, medical electricity, and practical work in the wards and physical exercises department, as well as in the electrical department.

The teaching staff is a singularly strong one; the medical superintendent, assisted by Dr. Mennell, who has made a special study of the treatment by massage of fractures under Dr. Championnière in Paris, gives lectures on the medical and surgical diseases capable of treatment by exercises and massage, as well as individual clinical teaching on patients, and electricity is taught in the same way also by a member of the staff. Miss Randell, the sister-in-charge, besides assisting at the lectures and classes given by the medical staff, is responsible for instructing the students in the technique of those different manipulations and movements which can only be acquired by months of laborious demonstration and practice. In addition to the very great advantages of individual teaching by expert medical men which the students enjoy, a great hospital such as St. Thomas's affords the very best and most varied clinical material, the cases for treatment averaging in a single week between 700 and 800, of which all women and children, and a proportion of the men, are dealt with by the lady students.

Having been taught theoretically, and having practised on each other the treatment likely to be ordered for surgical cases, such as wry neck, spinal curvature, or flat foot, or for medical ones, such as heart disease, pleurisy, or emphysema, the students are then allowed to visit the wards and apply the knowledge thus gained, under the direct supervision of a medical expert, learning how to modify the treatment for each individual case, and knowing that in difficult cases help and advice are always at hand. Perhaps the most interesting cases for which massage treatment is given are those of recent fractures and dislocations, and to anyone unacquainted with the great success which attends this treatment the sight of fractures in all stages, from the day of the accident onwards, is little short of a revelation. In the fracture-room may be seen injured limbs of all descriptions being manipulated and exercised under Dr. Meynell's direction, and evidently with the patient's cordial approval. The *x*-ray plate is carefully studied by the student before each treatment, and she is therefore able to know exactly what condition she is treating, and in all fractures which have been treated from the date of their occurrence remarkably successful results are demonstrated, deformity and impaired function being conspicuous by their absence, while union is complete within a third of the time usually allowed by the old system, a few familiar old Colles' fractures for which massage treatment had been ordered after other methods had failed, showing by comparison the great superiority of the "early treatment by massage system" to that of immobilisation.

Except for the want of room, which is certainly a drawback—for the premises, which appeared spacious when the school was opened, are now far too small—it is difficult to imagine more suitable conditions than are afforded to the students at this school for acquiring, in a most interesting and vivid way, an art which will provide them with a profitable, useful, and interesting occupation. Not least of the advantages offered by this school is the use of a dissecting-room for the study of practical anatomy. Any masseuse who reads this article will remember with what difficulty she acquired the knowledge of structures which she had never seen by means of diagrams and models, and will envy the lot of the St. Thomas's student, who has the same privilege of learning from an anatomy demonstrator on dissected parts as the medical student, while a course of lectures on elementary physics, mechanics, and electricity from the lecturer of the medical school on those subjects will prepare her for the medical electricity course, which is part of the equipment of a fully-trained masseuse.

